# Women with Suicidal Ideation, Substance Use Disorder, or Intimate Partner Violence in the Emergency Department: Retrospective Analysis of Contraceptive Documentation

**DOI:** 10.5811/westjem.48357

**Published:** 2025-12-23

**Authors:** Alison Ruch, Adam Henderson, Ania Izabela Rynarzewska, Hardeep Singh, Louise Jones

**Affiliations:** * Northeast Georgia Medical Center, Department of Emergency Medicine, Gainesville, Georgia; † Medical College of Georgia at Augusta University, Department of Emergency Medicine, Augusta, Georgia; ‡ Georgia College and State University, Department of Management, Marketing & Logistics Department, Milledgeville, Georgia; § Northeast Georgia Medical Center, Department of GME Research & Quality Improvement, Gainesville, Georgia

## Abstract

**Introduction:**

Prior research demonstrates that emergency department (ED) patients with suicidal ideation (SI), substance use (SUD), and/or intimate partner violence (IPV) have disproportionate adverse outcomes for both women and infants. The 2013 Hague Protocol suggested that children with caregivers with the above characteristics are also more likely to suffer from child maltreatment. Of all pregnancies in this group, as many as 90% are unintended. We hypothesized that women with SI/SUD/IPV have gaps in care access, high levels of unscheduled care use, and reduced ED contraceptive inquiry, which if addressed could potentially improve outcomes.

**Methods:**

We conducted a chart review of 62,284 ED visits from 2018–2021 from a suburban four-hospital system in the Southern United States. We compared women of reproductive age (15–44) with SI/SUD/IPV (4,776) against controls (57,508). The exposures were defined as women with SI, SUD, and/or IPV. We analyzed results using the chi-square test (χ^2^) with Bonferroni adjustment to test for independence and logistic regression.

**Results:**

Women suffering from SI/SUD/IPV who present to the ED have contraceptive status less frequently documented compared to controls without these factors (39.5 vs 51.7%, RR 0.77, CI, 0.74–0.79, P < .001). They also have reduced access to care, with higher rates of uninsurance (32.7 vs 26.1%, P < .001), more care in the acute care environment, longer ED length of stay (LOS) (mean was 10.38 vs 3.87 hours, P < .001), higher hospitalization rates (61.0 vs 8.7%, P < .001), and higher 30–day ED revisits (11.8 vs 8.8%, P < .001), even after adjusting for the Social Vulnerability Index, acuity, age, and obesity (adjusted odds ratio 1.52 95% CI 1.36–1.70 P < .001).

**Conclusion:**

Despite significant morbidity coupled with reduced access to ambulatory care and disproportionately increased ED use, little ED contraceptive documentation exists. This practice contributes to inequity, given the increased number of unintended pregnancies and greater need of contraceptives in women with suicidal ideation/substance use disorder/intimate partner violence.

## INTRODUCTION

Females suffering from suicidal ideation (SI), substance use disorder (SUD), and/or intimate partner violence (IPV) have disproportionately high mortality rates.^1^–^3^ In 2022, the National Safety Council reported 28,134 deaths in females from drug overdoses and, according to the US Centers for Disease Control and Prevention (CDC), in 2020–2021 there were 58,701 deaths of females attributable to alcohol use.^4^, ^5^ For women who survive a suicidal attempt at 20 years of age, life expectancy is shortened by 11 years.^6^ Over half of homicides in women are the result of by IPV, and about 75% of female IPV survivors experience some form of injury related to IPV.^7^ These conditions present at pre-conception can be predictive of the occurrence during pregnancy.^8^–^10^ Furthermore, as per the CDC, women with behavioral emergencies constitute the leading cause of pregnancy-related deaths in the US.^11^, ^12^

**Figure 1:**
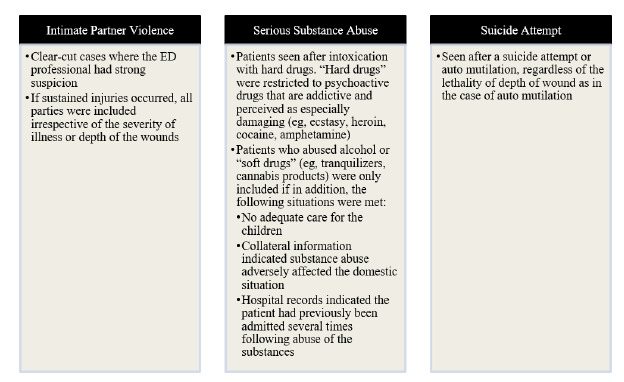
The Hague characteristics identify women suffering from suicidal ideation, substance use disorder, and/or intimate partner violence. *ED*, emergency department.

The consequences of IPV during pregnancy include an increased risk of pre-term birth, low birth weight, fetal injury, poor maternal mental health, and death.^13^ In SUD, increased rates of pre-term birth, stillbirth, fetal anomalies, and neurodevelopmental effects, as well as neonatal abstinence syndrome and fetal alcohol spectrum disorder can occur.^14^–^16^ Children of women with SI/SUD/IPV also experience higher rates of maltreatment and are at risk for adverse childhood experiences, which have been linked to chronic health problems.^17^, ^16^, ^18^ The 2013 Hague Protocol—based on a before-and-after study analyzing over one million caregiver visits to the ED—identified child maltreatment with 91% positive predictive value in children of adult ED patients who were found to have a serious psychiatric condition such as SI, SUD, or IPV during their ED course. (Figure 1).

Despite the deleterious outcomes for both mothers and infants, women with SI/SUD/IPV have rates of unintended pregnancies as high as 90%.^19^, ^20^–^22^ An unintended pregnancy is one that is unwanted or untimed.^23^ Unintended pregnancies are independently associated with increased adverse maternal and infant outcomes, including reduced antenatal care, child nutrition, child vaccination status, increased incidence of abortion, infant mortality, and poor maternal mental health conditions.^24^ In sum, maternal and infant mortality is high in SI/SUD/IPV, and pregnancies are more likely to be unintended in these behavioral patients. Most unintended pregnancies result from not using contraception or from not using it consistently or correctly.^23^ The American College of Obstetrics and Gynecology supports comprehensive and unhindered access to contraceptives, but there are currently no guidelines for the provision of comprehensive preventive family planning services in the ED.^24^–^26^

While it is unknown whether EDs may constitute an appropriate setting to offer preconception contraceptive services to populations with SI/SUD/IPV, mounting evidence supports the benefit of ED family planning interventions in targeted groups, such as pediatrics, or for those patients who lack access to family planning services.^27^–^29^ What is unique about SI/SUD/IPV patients is that they may have additional barriers to accessing traditional healthcare services and use the ED instead.^30^ For example in IPV, an abuser can isolate a woman and deprive her of money, transportation, and access to healthcare, or can sabotage efforts at contraception, refuse to practice safe sex, control the outcome of a pregnancy (by forcing a woman to continue the pregnancy, have an abortion, or to injure her in a way to cause a miscarriage), forbid sterilization, or control access to other reproductive health services.^31^,^32^ Ultimately, the ED remains a primary, discrete point of care for many at-risk patient groups.^33^

**Population Health Research Capsule**

What do we already know about this issue?

*Women with suicidal ideation (SI), substance use disorder (SUD), or intimate partner violence (IPV) face high maternal/infant mortality and poor access to reproductive care*.

What was the research question?

*Do women with SI/SUD/IPV in the emergency department (ED) have lower rates of contraceptive documentation than other women?*

What was the major finding of the study?

*Only 39.5% of SI/SUD/IPV women had contraception documented vs 51.7% controls (RR 0.77, CI, 0.74–0.79, P < .001)*.

How does this improve population health?

*Awareness of gaps in ED contraceptive care can aid emergency clinicians to better support interventions to reduce unintended pregnancy and improve outcomes for at-risk women*.

Additionally, access to primary care is significantly reduced among those with behavioral health emergencies.^34^, ^35^, ^36^, ^37^ In an effort to identify inequalities in care, our primary aim in this study was to evaluate differences in contraceptive documentation during ED visits between SI/SUD/IPV and non-SI/SUD/IPV groups. The secondary purpose was to evaluate the odds of women with SI/SUD/IPV having reduced odds of access to an established primary care physician (PCP) and increased use of unscheduled care as defined by ED length of stay (LOS), hospitalization rates, and ED re-visits.

## METHODS

This was an observational, retrospective chart review to assess contraceptive use by patients suffering from SI/SUD/IPV who visited a large, not-for-profit hospital system between 2018–2021. The system is comprised of four campuses in a suburban community setting in the Southern United States. Care settings included a non-university affiliated academic center and community centers that saw a combined annual patient population of approximately 200,000 patients in their EDs. The system’s main site has a Level I trauma center, a ST Elevation Myocardial Infarction (STEMI) center and a Comprehensive Stroke Center, and is accredited for pain and addiction care in the ED.

### Institutional Review Board Approval

The study received expedited approval from the institutional review board.

### Sampling Methods

**Figure 2:**
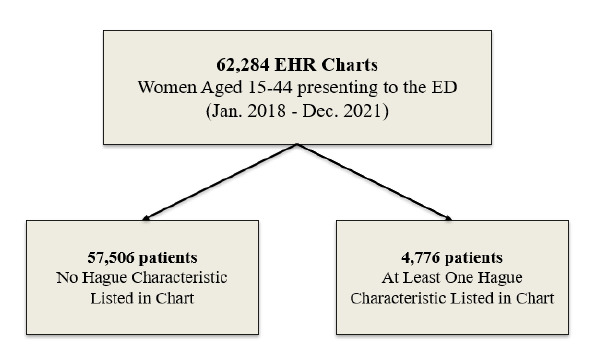
Women identified with the Hague characteristics of suicidal ideation, substance disorder and/or intimate partner violence compared to women presenting without Hague characteristics. *ED*, emergency department; *EHR*, electronic health record.

In this study we examined the charts from 62,284 women 15–44 years of age who initially presented to the ED between January 2018–December 2021. These encounters were sorted based on whether the patient had been in the ED with IPV, SI, and/or SUD. To test associations and differences between the groups on key characteristics and outcomes under investigation we compared women with no SI/SUD/IPV (56,708) to women without these characteristics (4,776) (Figure 2).

Based on previous literature, women with Hague characteristics were those who had SI/SUD/IPV. Missing data were retained, and we employed listwise deletion in the analysis. Chart data was de-identified using the safe harbor methodology and securely managed within a research data platform compliant with the Health Insurance Portability and Accountability Act. The historical data required to respond to research questions was fully de-identified before the investigation team received the final dataset fully anonymized.

### Case Selection Criteria

**Figure 3:**
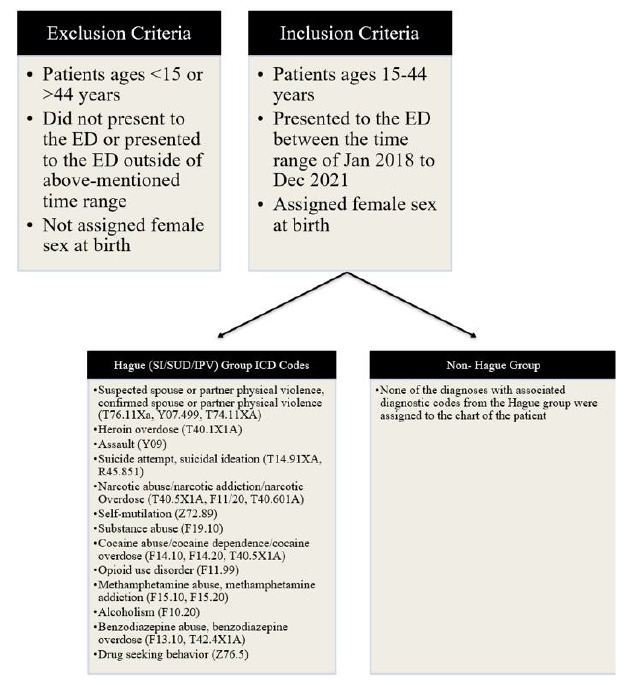
Inclusion and exclusion criteria for case groups comprised of women with and without suicidal ideation, substance use disorder and/or intimate partner violence who presented to the emergency department with specific diagnoses and respective *ICD-10 diagnostic codes included for the Hague group. *ICD-10*, International Classification of Diseases, 10^th^ Revision*; SI*, suicidal ideation; *SUD*, substance use disorder; *IPV*, intimate partner violence.

We included in the study women of reproductive age 15–44) with a lifetime ED visit with SI/SUD and/or IPV. Inclusion characteristics were defined by the *International Classification of Diseases, 10^th^ Revision* (*ICD-10*) (Figure 3) based on the Hague protocol.^17^ We specifically chose inclusion criteria based on the ED-specific Hague Protocol study,^17^ which enlisted caregiver risk factors that increased the vulnerability of their dependent children. Of note, marijuana use was not included in the *ICD-10* case selection criteria as the focus was to include conditions in the Hague protocol that were also associated with high mortality. As per the CDC, alcohol-attributable deaths in 2020–2021 were 178,307, and according to the National Safety Council, preventable drug overdoses in 2022 accounted for 99,592 deaths.^4^ Given the above, inclusion of cannabis would have artificially reduced the impact that substance use of hard drugs and alcohol has on outcomes and was, therefore, excluded.

### Variables Definitions

Each case was examined for SI/SUD/IPV (Figure 2) to determine group inclusion. Then, we compiled the following: the financial payor group; Social Vulnerability Index (SVI) score, social determinants of health (SDoH), discharge disposition,; length of time from arrival to ED to disposition from ED, ED re-visits rates (30 days); and presence of completed obstectrics/gynecology screening questions.

Uninsurance status was defined as lacking any type of insurance (Medicaid, Medicare, commercial, automobile, agency, veteran’s, or worker’s compensation), and these patients were self-pay. Having PCP care was defined as having an established PCP on record (typically, a family medicine or internal medicine practitioner, pediatrician, or gynecologist). Hospitalization included both psychiatric and transfers as well as medical admissions. Observation in the ED was excluded.

In addition to the variables of interest, we included SDoH, obesity, and age in the analysis, given their known negative impact on healthcare outcomes.^38^–^40^ The SDoH, in particular, are considered to be key factors affecting outcomes under investigation.^41^ Social vulnerabilities, including poverty, lack of access to transportation, and crowded housing, are directly related to SDoH.^42^ To account for the effect of social vulnerability, consistent with the CDC recommendations, we used the SVI, a measure of socioeconomic factors that affect how resilient communities are when facing disaster. In healthcare-related research, social vulnerability is used to determine its effects on health outcomes. The SVI comprises four key factors: socioeconomic status; household composition/disability; minority status/language; and housing/transportation factor (measured by housing structure, crowding, and vehicle access variables).^43^, ^44^

The SVI includes the following social factors: poverty (below 150% poverty estimate, 2018–2022 American Community Survey [ACS]; unemployment; having a minority status (Hispanic or Latino, Black, Asian, American Indian or Alaska Native, Native Hawaiian or Pacific Islander)^45^; housing cost burden (annual income < $75,000, which is 30%+ of income spent on housing costs, estimate of 2018–2022 ACS); non-high school diploma, uninsured, household total, > 65 years of age, < 17 years of age; disability; single-parent household; limited English proficiency; living in a multiunit house, mobile home, crowded living space or group quarters; and not having a vehicle. Each census tract received a flag if it was in the 90% for the metric.

We defined obesity as a body mass index ≥ 30. A 30-day repeat ED visit was defined as repeat ED encounters in a span of 30 days. Acuity was defined by Emergency Severity Index (ESI) level, with ESI 1 being highest and ESI 5 lowest. We defined contraceptive status as a method of contraception, including no method documented, abstinence, condom, chemo/radiation, ablation, postmenopausal, perimenopausal, pregnant or recently pregnant, oophorectomy, injection, intrauterine device, implant, oral contraceptive, hysterectomy, or tubal ligation. No formal requirement for contraceptive documentation is currently part of the ED nursing triage system; thus, any contraceptive documentation that did occur was likely either previously documented (from other visits), documented during the ED visit on a case-by-case basis, or documented during the follow-up period at the moment in which the data was collected, We defined ED LOS as the time from ED intake to ED discharge from the facility or to the next point of care.

### Medical Record Identified

Project data from the hospital’s clinical research data platform Epic (Epic Systems Corporation, Verona, WI) was collected by the blinded data administrator of graduate medical education, in compliance with both institutional and federal requirements.

### Abstractor Training

The primary abstractors were trained and/or certified in the use of the data analytics platform Qlik Sense (QlikTechnologies Inc., King of Prussia, PA) and had data architect and business analyst certifications or training.

### Abstractor Forms

Data abstractor forms were used to define the variables and inclusion/exclusion criteria.

### Abstractor Blinding

The abstractors were part of this or additional research studies for this team and, therefore, had previously been introduced to the hypothesis and study objectives. However, the data were de-identified after being collected from the database based on 45CFR 46 guidelines of the US Department of Health & Human Services.^46^

### Performance Monitoring and Interobserver Reliability

Approximately 10% of the data for patients in the SI/SUD/IPV group were reviewed manually by the data developer and first author to confirm accurate data inclusion and exclusion (age, sex, and an ED visit with IPV and/or a severe behavioral health concern). A qualitative measure determined interobserver reliability. It was based on an agreement between both abstractors.^47^

### Missing Data Management Plan

Statistics for each category were based on all the cases with valid data in the specified ranges for all variables in the categories. Of the 62,284 sample size, 800 charts (1.3%) did not have a Hague characteristic documented and were excluded from analysis using the listwise deletion approach, which is standard practice when dealing with missing data below a 5% threshold.^48^ If no contraceptive was documented, it was coded as “contraceptive not documented,” which resulted in 49.3% of all included women (30,294) not having contraception status documented. If Epic had multiple forms of contraception documented, the notes and data entry points were reviewed for accuracy, and all forms of contraception were included in the analysis and coded as “contraception documented.”

As the study was retrospective in nature, no causal relation can be established. We attempted to establish the odds of certain outcomes. In doing so, our goal was that the model would predict the odds of deleterious outcomes occurring based on the variables studied.

Research questions and the level of measurement suggested the appropriate statistical test. We analyzed data using SPSS 28 (IBM Corporation, Armonk, NY). To determine whether a statistical association existed between women with Hague characteristics vs non-Hague characteristics in terms of odds of contraceptive documentation, and due to the nominal nature of the variables, we conducted a chi-square test of independence with Bonferroni adjustment for a post hoc test. To test for differences in the LOS between women with and without SI/SUD/IPV characteristics, we used an independent sample *t*-test. Finally, to determine the odds of contraceptive documentation, 30-day revisits, and hospitalization, we used a logistic regression. Missing cases were treated using listwise deletion.

As described in this section, we followed established guidelines for reporting observational studies.^38^ To summarize what has been described in this section, the following optimal retrospective chart review methods, as defined by Worster et al,^39^ were followed to the extent that was possible and were outlined for compliance purposes as subtitles above.

## RESULTS

Table 1 represents the differences in demographics and insurance types between women with and without SI/SUD/IPV characteristics, as well as the number of women in each sub-category (SI/SUD/IPV). Women with suicidal ideation were the highest subset of the case sample, followed by SUD and IPV. Women with multiple characteristics comprised the smallest, although still substantial, subsample (see Table 1).

A 2x2 chi-square cross-tabulation test suggests that the contraceptive status of women with SI/SUD/IPV was less frequently documented (39.5 vs 51.7%, *P* < .001) with lower relative risk (RR) for contraceptive status reporting in SI/SUD/IPV women (RR 765, 95% CI, .74 – .79). This remained true (aOR .54, 95% CI, .50 – .58, *P* <.001) even after adjusting for SVI status, acuity, and ED LOS in logistic regression analysis. Further, based on the logistic regression analysis that adjusted for the presence of SVI, acuity, and the LOS, the odds of contraceptive documentation varied based on acuity and LOS in the non-Hague group only. Indeed, low emergency severity indices (ESIs) of 4 and 5 have an aOR of contraceptive documentation of 0.72 (CI 0.53. – 0.97, *P* <.05) and 0.60 (CI, 0.42 – 0.85, *P* <.01), respectively. Emergency department LOS had an aOR of 1.02 (CI, 1.01–1.02, *P* <.001) for contraceptive documentation. However, none of these factors affected the documentation odds of women with Hague characteristics. In these women, no statistically significant trend was associated with acuity or LOS (see Table 2).

Table 2 demonstrates that women with Hague characteristics had contraceptive status less frequently documented (39.5 vs 51.7%, *P* < .001) with lower RR for contraceptive status reporting in SI/SUD/IPV women (RR .765, 95% CI, .74 – .79). This remained true (aOR .54, 95% CI, .50–.58, *P* <.001) even after adjusting for SVI status, acuity, and ED LOS in logistic regression analysis. Further, based on the logistic regression analysis that adjusted for the presence of SVI, acuity, and the LOS, the odds of contraceptive documentation varied based on acuity and LOS in the non-Hague group only. Low ESIs of 4 and 5 had an aOR of contraceptive documentation of 0.72 (CI, 0.53 – 0.97, *P* <.05) and 0.60 (CI, 0.42 – 0.85, *P* <.01), respectively. Emergency department LOS had an aOR of 1.02 (CI, 1.01–1.02, *P* <.001) for contraceptive documentation. However, none of these factors affected SI/SUD/IPV women’s documentation odds. In women with Hague characteristics, no statistically significant trend was associated with acuity or LOS.

Women with SI/SUD/IPV who presented to the ED experienced lower rates of PCP care (50 vs 46.1%, *P* <.001) and higher rates of uninsurance (32.7 vs 26.1%, *P* <.001). Relative risk for being uninsured was higher for these women (RR 1.25, 95% CI, 1.20–1.31), as was the RR of not having a PCP (RR 1.19, 95% CI, 1.15–1.22). Women in the Hague category also had longer ED LOS (a mean of 10.38 hours vs 3.87 hours, mean difference 6.5, 95% CI, −6.8, −6.2, *P* <.001). Results from a logistic regression and chi-square test of independence with Bonferroni adjustment for post hoc analysis indicate that women with SI/SUD/IPV had higher rates of hospitalization (61 vs 8.7%, *P* <.001) and significantly higher odds of being hospitalized (aOR 17.02, CI, 15.87–18.25, *P* <.001) even after adjusting for SVI, age, and obesity (see Table 3).

Table 3 demonstrates that women presenting with Hague characteristics had higher rates of hospitalization (61 vs 8.7%, *P* < .001) and had significantly higher odds of being hospitalized (aOR 17.02, CI, 15.87–18.25, *P* < .001) even after adjusting for SVI, age, and obesity. Further, women with Hague characteristics had higher rates of 30-day ED-repeat visits (11.9 vs 8.8%, aOR 1.52, 95% CI, 1.36–1.70, *P* <. .001) even after adjusting for SVI, acuity, age, and obesity (see Table 4).

Table 4 demonstrates that women with Hague characteristics had higher rates of 30-day ED-repeat visits (11.9 vs 8.8%, aOR. 1.52, 95% CI, 1.36–1.70, *P* < .001) even after adjusting for SVI, acuity, age, and obesity.

## DISCUSSION

A high prevalence of SI/SUD/IPV characteristics, which are considered by the CDC as representing vulnerability, require attention to ensure that the population of women with these characteristics are adequately served in ED.^49^ However, results from this large, multicenter, retrospective cohort study highlight that women with SI/SUD/IPV emergencies had reduced contraceptive documentation, regardless of a woman’s acuity or ED LOS, which suggests that this particular population of women is underserved. These women were additionally associated with reduced access to care, high ED use rates, and increased hospitalizations. This is the first study to evaluate non-emergency contraceptive inequalities in the ED among patients with behavioral characteristics of SI/SUD/IPV. Less documentation may reflect reduced access to family planning services due to clinicians’ fear of being perceived as judgmental, or as a result of stigmatization of these women or increased incidence of physician burnout associated with their complex social and behavioral needs.^50^ Emergency department literature is limited, as prior studies have excluded behavioral patients and patients with emotional distress who might still desire (and benefit) from family planning services.^51^,^28^ In one such study (which excluded behavioral patients), emergency clinicians only asked about contraceptive use 23% of the time, over 90% “never” provided condoms, injections, implants, or IUDs, and 60% reported “never” providing prescriptions for non-emergency contraceptive pills, patches, or rings.^52^ Our study highlights that the odds were likely even lower in behavioral patients. Additionally, the reduced access to care and reduced contraceptive documentation demonstrated in this study is in line with prior research demonstrating a link between improved access to family planning with insurance expansion.^53^

Consistent with prior literature, this study’s results support the notion that women suffering from SI/SUD/IPV are known to be more likely to be frequent ED users.^54^,^55^ This study demonstrated frequent ED use, ED LOS, hospitalization, and ED re-visits. Given that these women interface with acute care clinicians at high rates and have significant unmet care needs, EDs could bridge a care gap and provide targeted interventions to improve access to contraceptive counseling and initiation in those patients who desire such services.^29^,^56^–^58^ Current non-emergency screening and preventative services routinely carried out in some EDs include targeted basic pharmacologic coverage (such as non-emergent medications like antihypertensives), vaccination programs, and hepatitis C and HIV screenings.^51^,^59^–^62^

Reducing barriers to contraception initiation (to include initiating at the time when a woman requests) has been recognized as an important initiative to reduce unintended pregnancies.^63^ Integrating care in populations with SI/SUD/IPV is increasingly supported.^64^–^66^ This study highlights that women with SI/SUD/IPV have an opportunity to receive family planning services due to the increased time they spend in the acute care setting. We suspect that lack of contraceptive documentation reflects both the reduced access to outpatient family planning services that behavioral patients face and the lack of non-emergent contraceptive counseling that occurs in the ED. Indeed, there has been little emphasis in emergency medicine training and practice on preventive contraceptive counseling and initiation from the ED, and placement of an etonogestrel implants, for example, requires additional training and certification.^67^,^68^

Given the limited follow-up care in these populations, effective programs that can both address the underlying risk factors while bridging the family planning gaps may be developed. For example, in an ED patient with opioid use disorder who is at risk of an unwanted pregnancy, treatment of the underlying SUD could be initiated along with contraceptive initiation. In the state of Georgia where this study was conducted, trained registered nurses may place a contraceptive injectable or an implant.^69^ In this way, a patient could receive care for her underlying SUD and address modifiable SDoH, as well as bridge the contraceptive gap, if desired, while she embarks on the road to recovery. By initiating a long-acting contraception, less urgent follow-up would be required, and the ED could orchestrate links to accessible community programs that match the patient’s needs.

The results of this study highlight an opportunity for further research to address an ED intervention to reduce the adverse outcomes of unintended pregnancies, increased rates of unintended pregnancies in populations with mental health issues, and the long-lasting increased rates of adverse childhood experiences in the offspring of women suffering from SI/SUD/IPV. With this gap, a potential opportunity to improve the outcomes of both women and their children by addressing non-emergency contraceptive care in the ED may be on the horizon. This study provides evidence that highlights the inequity in family planning services in ED patients with SI/SUD/IPV and, therefore, an unmet need and opportunity. The American College of Obstetrics and Gynecology supports funding for research that identifies effective strategies to reduce health inequities in unintended pregnancy and access to contraception.^70^ Future studies should evaluate and include women with behavioral risk factors and clinicians’ perspective and barriers to contraceptive initiation in the ED, patients’ preferred method(s), and patient-centered, non-coercive successful models that demonstrate how to operationalize these services in the post-COVID-19 era of boarding and ED crowding.

## LIMITATIONS

Despite numerous strengths of this research that focuses on the understudied and potentially underserved population of women, this cohort study had several limitations. First, it was conducted in a single hospital system with a patient demographic and access to services that may not represent the rest of the country. Second, while equal sub-populations in a study such as this should not be expected, it needs to be mentioned that the proportion of patients with SI/SUD/IPV was not equally distributed. Further, patients with IPV are often under-reported, which could have resulted in lower incidence of IPV reported or the quantity of multiple inclusion characteristics reported. The *I*C*D-10* codes used to screen for Hague populations may have missed inclusion characteristics that were not identified by those codes. Additionally, the control group was significantly larger than the case group, although this did not per se affect the outcome in the analysis conducted.

It is possible that contraceptive discussions were performed, but not documented, due to multiple factors including lack of reimbursement associated with reproductive counseling in the ED or the lack of perceived relevance by ED staff. It is also possible that the documentation present was from those established with the system (and PCP) and not because contraceptive care was discussed in the ED, as the system in which this study was performed does not currently have protocolized contraceptive care in the ED. Additionally, data in the study overlap with data collected at the beginning of the COVID-19 pandemic, which may have affected how patients used the ED. Another study suggested that mental health ED visits increased during the COVID-19 pandemic,^71^ which could have disproportionally worsened the use of the acute care setting by behavioral patients, thus skewing the results of this study. Nevertheless, a possible disproportionate increase in the susceptibility of deleterious outcomes in behavioral populations would heighten the importance of addressing the disparities present in the care of these women.

Despite the limitations of this study, its contribution to the literature is of value given the scarcity of research focusing on family planning in the ED and the significant inequity in the reproductive outcomes of behavioral women. Therefore, we believe the data from this study can provide a background framework that can highlight opportunities to improve care in these populations.

## CONCLUSION

Women of reproductive age who struggle with suicidal ideation, substance use disorder, and or intimate partner violence suffer from significant reproductive service inequality, which contributes to inequity for both the mothers and their children. This research highlights that women who suffer from SI/SUD/IPV have less contraceptive status documentation, fewer established primary care physicians, and higher rates of uninsurance, which likely limit their access to preventive contraception despite their increased need. Despite deleterious maternal outcomes, childhood-related adverse outcomes, elevated rates of unintended pregnancies in these populations, and reduced access to ambulatory care, these women are not receiving care in the ED that could bridge their care gaps.

While the ED can be fast paced and perceived to have insufficient time to address family planning, this study highlights that women with SI/SUD/IPV characteristics had lower prevalence and odds of having their contraceptive status documented. At the same time, this study documents that these patients have longer ED LOS, higher rates of hospitalization, and higher rates of 30-day ED-revisits. This increased contact time with the acute care setting may allow emergency clinicians to identify care gaps in reproductive healthcare and potentially improve the outcomes of marginalized women and their children with interventions. Further research to understand these patients and gauge clinicians’ interest in ED contraceptive counseling and initiation in the ED, in addition to contraceptive service preferences, barriers, and patient-centered processes that are inclusive should be prioritized.

## Figures and Tables

**Table 1:** Demographics and insurance type of women with and without characteristics of suicidal ideation, substance use disorder, and/or intimate partner violence who presented to the emergency. department.

		Non-SI/IPV/SUD	SUD	Suicidal	IPV	Multi	Total
Insurance	Commercial	2,4672	359	906	119	91	26,147
		48.1%	19.7%	45.9%	26.7%	27.4%	46.8%
	Medicaid	11,898	557	635	147	99	13,336
		23.2%	30.6%	32.2%	33.0%	29.8%	23.9%
	Medicare	1,315	76	61	11	16	1,479
		2.6%	4.2%	3.1%	2.5%	4.8%	2.6%
	Self-pay	13,378	831	370	169	126	14,874
		26.1%	45.6%	18.8%	37.9%	38.0%	26.6%
	Total	51,263	1,823	1,972	446	332	55,836
Patient ethnicity	Not Hispanic	45,532	1,781	1,752	391	328	49,784
		81.4%	95.3%	86.1%	83.0%	94.8%	82.0%
	Hispanic	10,436	87	284	80	18	10,905
		18.6%	4.7%	13.9%	17.0%	5.2%	18.0%
	Total	55,968	1,868	2,036	471	346	60,689
Patient race	Unknown	577	15	27	1	4	624
		1.0%	0.8%	1.3%	0.2%	1.1%	1.0%
	White	37,861	1,653	1,541	315	302	41,672
		66.8%	87.4%	74.7%	66.5%	86.5%	67.8%
	Black	8,132	143	224	80	27	8,606
		14.3%	7.6%	10.9%	16.9%	7.7%	14.0%
	Asian	724	3	27	3	0	757
		1.3%	0.2%	1.3%	0.6%	0.0%	1.2%
	Other	9,414	77	243	75	16	9,825
		16.6%	4.1%	11.8%	15.8%	4.6%	16.0%

*SI*, suicidal ideation; *SUD*, substance use disorder; *IPV*, intimate partner violence.

**Table 2:** Contraceptive documentation in the emergency department for women with suicidal ideation, substance use disorder, and/or intimate partner violence.

	All Women				Non-Hague status				Hague characteristics (SI/SUD/IPV)			
		
	Sig. *P*-value	aOR	95% CI for aOR		Sig. *P*-value	aOR	95% CI for aOR		Sig. P-value	aOR	95% CI for aOR	
		
			Lower	Upper			Lower	Upper			Lower	Upper
SVI Total	>.05	1.01	1.00	1.01	>.05	1.01	1.00	1.02	> .05	0.99	0.96	1.02
ED LOS (hours)	< .001	1.01	1.01	1.01	< .001	1.02	1.01	1.02	> .05	1.00	1.00	1.01
ESI 1 (immediate)	< .001	Reference			< .001	reference			< .05	reference		
ESI 2 (emergent)	>.05	1.12	0.86	1.47	> .05	1.20	0.88	1.64	> .05	1.01	0.57	1.77
ESI 3 (urgent)	<.05	1.33	1.02	1.74	< .05	1.38	1.02	1.86	> .05	1.29	0.73	2.28
ESI 4 (less urgent)	<.01	0.69	0.52	0.90	< .05	0.72	0.53	0.97	> .05	1.09	0.57	2.07
ESI 5 (non-urgent)	< .001	0.57	0.41	0.78	< .01	0.60	0.42	0.85	> .05	0.00	0.00	
SI/SUD/IPV Status^1^	< .001	0.54	0.51	0.59	NA				NA			
Obesity^1^	> .05	1.15	0.62	2.16	> .05	1.26	0.63	2.49	p>.05	0.41	0.05	3.64

Contraceptive status documentation as an outcome, with multiple variables (SVI, ED LOS, ESI, Hague status, and obesity) that may contribute to such.

1 defined as the presence of the noted variable.

*aOR*, adjusted odds ratio; *ED*, emergency department; *ESI*, Emergency Severity Index. *LOS*, length of stay; *NA*, not applicable; *SI*, suicidal ideation; *SUD*, substance use disorder; *IPV*, intimate partner violence; *SVI*, Social Vulnerability Index.

**Table 3:** Rates of hospitalization in women with suicidal ideation, substance disorder, and/or intimate partner violence who presented to the emergency department.

	Wald	Sig. *P*-value	aOR	95% CI for aOR	

				Lower	Upper
SI/SUD/IPV status^1^	6306.9	<.001	17.02	15.87	18.25
Obesity^1^	21.7	<.001	4.83	2.49	9.36
SVI total	0.0	>.05	1.00	0.99	1.01
Age	145.3	< .001	1.02	1.02	1.02

1 presence of the noted variable.

*aOR*, adjusted odds ratio *SI*, suicidal ideation; *SUD*, substance use disorder; *IPV*, intimate partner violence; *SVI*, Social Vulnerability Index.

**Table 4:** 30- day emergency department re-visits for women with suicidal ideation, substance disorder, and/or intimate partner violence.

	Wald	Sig. *P*-value	aOR	95% CI for aOR	

				Lower	Upper
SVI total	24.99	< .001	1.04	1.02	1.05
ESI 1 (immediate)	11.83	< .05	reference		
ESI 2 (emergent)	0.00	> .05	0.99	0.62	1.57
ESI 3 (urgent)	0.27	> .05	1.13	0.71	1.80
ESI 4 (less urgent)	0.06	> .05	1.06	0.67	1.69
ESI 5 (Non Urgent)	1.41	> .05	1.38	0.81	2.37
SI/SUD/IPV status^1^	52.83	<.001	1.52	1.36	1.70
Obesity^1^	0.00	> .05	1.02	0.36	2.85
Age	25.445	< .001	1.01	1.01	1.01

1 presence of the noted variable.

*aOR*, adjusted odds ratio; *ESI, Emergency Severity Index; SVI*, Social Vulnerability Index.

## References

[1] ARDI Alcohol-Attributable Deaths.

[2] Aldridge RW, Story A, Hwang SW (2018). Morbidity and mortality in homeless individuals, prisoners, sex workers, and individuals with substance use disorders in high-income countries: a systematic review and meta-analysis. The Lancet.

[3] Jack SPD (2015). Surveillance for Violent Deaths — National Violent Death Reporting System, 27 States. MMWR Surveill Summ.

[4] ARDI Alcohol-Attributable Deaths.

[5] Drug Overdoses - Data Details. Injury Facts.

[6] Jokinen J, Talbäck M, Feychting M (2018). Life expectancy after the first suicide attempt. Acta Psychiatr Scand.

[7] Jack SPD, Petrosky E, Lyons BH (2018). Surveillance for Violent Deaths - National Violent Death Reporting System, 27 States, 2015. Morb Mortal Wkly Rep Surveill Summ Wash DC 2002.

[8] WHO multi-country study on women’s health and domestic violence against women: summary report.

[9] Albright BB, Rayburn WF (2009). Substance abuse among reproductive age women. Obstet Gynecol Clin North Am.

[10] Legazpi PCC, Rodríguez-Muñoz MF, Le HN (2022). Suicidal ideation: prevalence and risk factors during pregnancy. Midwifery.

[11] (2016). CDC Newsroom.

[12] Shigemi D, Ishimaru M, Matsui H (2021). Suicide attempts during pregnancy and perinatal outcomes. J Psychiatr Res.

[13] Agarwal S, Prasad R, Mantri S (2023). A Comprehensive review of intimate partner violence during pregnancy and its adverse effects on maternal and fetal health. Cureus.

[14] CDC (2025). Substance Use During Pregnancy.

[15] Dejong K, Olyaei A, Lo JO (2019). Alcohol use in pregnancy. Clin Obstet Gynecol.

[16] CDC (2025). About Adverse Childhood Experiences Adverse Childhood Experiences (ACEs).

[17] Diderich HM, Fekkes M, Verkerk PH (2013). A new protocol for screening adults presenting with their own medical problems at the emergency department to identify children at high risk for maltreatment. Child Abuse Negl.

[18] CDC (2021). Preventing Adverse Childhood Experiences.

[19] Black KI, Stephens C, Haber PS (2012). Unplanned pregnancy and contraceptive use in women attending drug treatment services. Aust N Z J Obstet Gynaecol.

[20] Heil SH, Jones HE, Arria A (2011). Unintended pregnancy in opioid-abusing women. J Subst Abuse Treat.

[21] Jones HE, Berkman ND, Kline TL (2011). Initial feasibility of a woman-focused intervention for pregnant African-American women. Int J Pediatr.

[22] Martin-de-las-Heras S, Velasco C, Luna J, de D (2015). Unintended pregnancy and intimate partner violence around pregnancy in a population-based study. Women Birth J Aust Coll Midwives.

[23] CDC (2025). Unintended Pregnancy. Reproductive Health.

[24] Providing Quality Family Planning Services: Recommendations of CDC and the U.S. Office of Population Affairs.

[25] Emergency Reproductive Health.

[26] Family Planning.

[27] Caldwell MT, Choi H, Levy P (2018). Effective contraception use by usual source of care: an opportunity for prevention. Womens Health Issues.

[28] Caldwell MT, Hambrick N, Vallee P (2020). “They’re doing their job”: women’s acceptance of emergency department contraception counseling. Ann Emerg Med.

[29] Liang AC, Sanders NS, Anderson ES (2023). “ContraceptED”: a multidisciplinary framework for emergency department-initiated contraception. Ann Emerg Med.

[30] Richardson LD, Hwang U (2001). Access to care: a review of the emergency medicine literature. Acad Emerg Med.

[31] (1992). American Medical Association diagnostic and treatment guidelines on domestic violence. Arch Fam Med.

[32] Reproductive health and partner violence guidelines: an integrated response to intimate partner violence and reproductive coercion. VAWnet.org.

[33] Selby S, Wang D, Murray E (2018). Emergency departments as the health safety nets of society: a descriptive and multicenter analysis of social worker support in the emergency room. Cureus.

[34] Benjamin-Johnson R, Moore A, Gilmore J (2009). Access to medical care, use of preventive services, and chronic conditions among adults in substance abuse treatment. Psychiatr Serv Wash DC.

[35] Kilbourne AM, McCarthy JF, Post EP (2006). Access to and satisfaction with care comparing patients with and without serious mental illness. Int J Psychiatry Med.

[36] Saloner B, Bandara S, Bachhuber M (2017). Insurance coverage and treatment use under the affordable care act among adults with mental and substance use disorders. Psychiatr Serv Wash DC.

[37] Btoush R, Campbell JC, Gebbie KM (2008). Visits coded as intimate partner violence in emergency departments: characteristics of the individuals and the system as reported in a national survey of emergency departments. J Emerg Nurs.

[38] CDC (2025). How Overweight and Obesity Impacts Your Health. Healthy Weight and Growth.

[39] Mode NA, Evans MK, Zonderman AB (2016). Race, neighborhood economic status, income inequality and mortality. Olson DR, ed. PLoS ONE.

[40] Committee on the Health and Medical Dimensions of Social Isolation and Loneliness in Older Adults, Board on Health Sciences Policy, Board on Behavioral, Cognitive, and Sensory Sciences, Health and Medicine Division, Division of Behavioral and Social Sciences and Education, National Academies of Sciences, Engineering, and Medicine (2020). Social Isolation and Loneliness in Older Adults: Opportunities for the Health Care System.

[41] CDC Social Determinants of Health (2025). Health Disparities in HIV, Viral Hepatitis, STDs, & Tuberculosis.

[42] CDC (2024). SVI Fact Sheet Place and Health - Geospatial Research, Analysis, and Services Program (GRASP).

[43] Flanagan BE, Gregory EW, Hallisey EJ A social vulnerability index for disaster management. https://stacks.cdc.gov/view/cdc/134506.

[44] Social vulnerability indices: a scoping review. BMC Public Health Full Text.

[45] (2025). Race and National Origin.

[46] (2016). Protections (OHRP) O for HR. 45 CFR 46.

[47] (2024). Inter-rater reliability methods in qualitative case study research - Rosanna Cole.

[48] Marino M, Lucas J, Latour E (2021). Missing data in primary care research: importance, implications and approaches. Fam Pract.

[49] Swedo EA (2024). Adverse childhood experiences and health conditions and risk behaviors among high school students — youth risk behavior survey, United States, 2023. MMWR Suppl.

[50] Cho HL, Huang CJ (2020). Why mental health-related stigma matters for physician wellbeing, burnout, and patient care. J Gen Intern Med.

[51] Liles I, Haddad LB, Lathrop E (2016). Contraception initiation in the emergency department: a pilot study on providers’ knowledge, attitudes, and practices. South Med J.

[52] Ketterer T, Sieke E, Min J (2024). Contraception initiation in the emergency department: adolescent perspectives. J Adolesc Health.

[53] Moniz MH, Kirch MA, Solway E (2018). Association of access to family planning services with Medicaid expansion among female enrollees in Michigan. JAMA Netw Open.

[54] Moulin A, Evans EJ, Xing G (2018). Substance use, homelessness, mental illness and Medicaid coverage: a set-up for high emergency department utilization. West J Emerg Med.

[55] Bonomi AE, Anderson ML, Rivara FP (2009). Health care utilization and costs associated with physical and nonphysical-only intimate partner violence. Health Serv Res.

[56] Preiksaitis C, Henkel A (2023). The evolving role of emergency medicine in family planning services. Curr Opin Obstet Gynecol.

[57] Gutman CK, Dorfman D, Meese H (2020). Identifying a golden opportunity: adolescent interest in contraceptive initiation in a pediatric emergency department. J Womens Health 2002.

[58] Miller MK, Goggin K, Stancil SL (2024). Feasibility of adolescent contraceptive care in the pediatric emergency department: a pilot randomized controlled trial. Acad Emerg Med.

[59] Brody A, Rahman T, Reed B (2015). Safety and efficacy of antihypertensive prescription at emergency department discharge. Acad Emerg Med.

[60] Rimple D, Weiss SJ, Brett M (2006). An emergency department-based vaccination program: overcoming the barriers for adults at high risk for vaccine-preventable diseases. Acad Emerg Med.

[61] Hsieh YH, Rothman RE, Laeyendecker OB (2016). Evaluation of the Centers for Disease Control and Prevention recommendations for hepatitis C virus testing in an urban emergency department. Clin Infect Dis.

[62] Spagnolello O, Reed MJ (2021). Targeted HIV screening in the emergency department. Intern Emerg Med.

[63] US Selected Practice Recommendations for Contraceptive Use, 2016.

[64] MacAfee LK, Harfmann RF, Cannon LM (2020). Substance use treatment patient and provider perspectives on accessing sexual and reproductive health services: barriers, facilitators, and the need for integration of care. Subst Use Misuse.

[65] (2012). ACOG Committee Opinion No. 518: Intimate partner violence. Obstet Gynecol.

[66] Ko JY (2017). CDC Grand Rounds: public health strategies to prevent neonatal abstinence syndrome. Morb Mortal Wkly Rep.

[67] CORD Appendix C Emergency Medicine Goals and Objectives.

[68] Request Clinical Training.

[69] McConnell A (2023). Rural Georgia gets more contraceptive options through expanded delivery of birth control implants. Georgia Recorder.

[70] (2015). Committee Opinion No. 615: Access to Contraception. Obstet Gynecol.

[71] Mojtahedi Z, Guo Y, Kim P (2023). Mental health conditions- and substance use-associated emergency department visits during the COVID-19 pandemic in Nevada, USA. Int J Environ Res Public Health.

